# Semaphorin 3A controls enteric neuron connectivity and is inversely associated with synapsin 1 expression in Hirschsprung disease

**DOI:** 10.1038/s41598-020-71865-3

**Published:** 2020-09-15

**Authors:** Jacques Gonzales, Catherine Le Berre-Scoul, Anne Dariel, Paul Bréhéret, Michel Neunlist, Hélène Boudin

**Affiliations:** 1 Inserm UMR1235-TENS, University of Nantes, Inserm, TENS, The Enteric Nervous System in Gut and Brain Diseases, IMAD, 1 rue Gaston Veil, 44035 Nantes, France; 2 Present Address: Pediatric Surgery Department, Hôpital Timone-Enfants, Assistance Publique des Hôpitaux de Marseille, Marseille, France

**Keywords:** Developmental biology, Neuroscience, Gastroenterology

## Abstract

Most of the gut functions are controlled by the enteric nervous system (ENS), a complex network of enteric neurons located throughout the wall of the gastrointestinal tract. The formation of ENS connectivity during the perinatal period critically underlies the establishment of gastrointestinal motility, but the factors involved in this maturation process remain poorly characterized. Here, we examined the role of Semaphorin 3A (Sema3A) on ENS maturation and its potential implication in Hirschsprung disease (HSCR), a developmental disorder of the ENS with impaired colonic motility. We found that Sema3A and its receptor Neuropilin 1 (NRP1) are expressed in the rat gut during the early postnatal period. At the cellular level, NRP1 is expressed by enteric neurons, where it is particularly enriched at growth areas of developing axons. Treatment of primary ENS cultures and gut explants with Sema3A restricts axon elongation and synapse formation. Comparison of the ganglionic colon of HSCR patients to the colon of patients with anorectal malformation shows reduced expression of the synaptic molecule synapsin 1 in HSCR, which is inversely correlated with Sema3A expression. Our study identifies Sema3A as a critical regulator of ENS connectivity and provides a link between altered ENS connectivity and HSCR.

## Introduction

In the nervous system, the formation of neuronal circuits is controlled by a large repertoire of molecules acting in a coordinated manner on axonal elongation and synapse formation.The enteric nervous system (ENS) is part of the peripheral nervous system contained within the gut wall that integrates local and systemic signals to control gut motility, secretion, intestinal permeability and epithelial cell proliferation^1^. The ENS is composed of enteric neurons and glial cells assembled in ganglia interconnected through extensive longitudinal and circumferential axonal arborization. Enteric neurons and glia are derived from precursors that originate in the neural crest, migrate to the gut, and undergo a complex process of developmental maturation extending to the postnatal period and resulting in functional maturation^2,3^. While the mechanisms involved in the migration of ENS progenitor cells and their differentiation into neurons and glia have been widely studied^2,4–7^, much less is known about how enteric neurons establish connections to form a mature neuronal network^8–10,14^. It is therefore critical to delineate the mechanisms involved in the formation of the axonal processes that ultimately dictate neuronal connectivity and ENS functions. Evidence indicated that cell migration and axonal outgrowth share common molecular mechanisms in the developing ENS, such as those involving GDNF or sonic hedgehog^15–17^. In particular, the temporal regulation of GDNF expression, together with its diversity of the cellular sources throughout ENS development are thought to underlie the multiple functions attributed to GDNF on neural crest cell migration, neuronal differentiation, neurite extension and synaptogenesis^7,11,18,19^. Semaphorin 3A (Sema3A), a prototypical class 3 secreted semaphorin, could also represent a molecule with multifaceted roles throughout ENS development because such a diversity of Sema3A activities have been extensively demonstrated in the central nervous system^20^.

Sema3A controls the migration of cortical neurons^21^ and is implicated in axon repulsion, dendritic branching and synapse formation of central neurons via binding with its receptor Neuropilin 1 (NRP1)^20,22^. In the ENS, Sema3A has been shown to regulate the entry of sacral enteric neural precursors into the distal hindgut in mice by acting as a repulsive signal secreted from the intestine mesenchyme^23^. Whether Sema3A could also regulate axonal patterning and connectivity of the ENS is currently unknown. Moreover, the expression and distribution of Sema3A and its obligatory receptor NRP1 have never been studied in the ENS. Interestingly, changes in Sema3A expression have been described in the colon of some patients with Hirschsprung disease (HSCR) and rare coding and non-coding deleterious variants of Sema3A have been identified in HSCR cohorts^24–26^. HSCR is a multifactorial genetic developmental disorder in which the ENS is missing from distal bowel resulting in a tonic contraction of the segment leading to functional obstruction. The only current treatment is the operative removal of the segment devoid of ENS. However, recent studies suggested that the remaining bowel, although considered healthy, presents also ENS defects such as altered proportion of nitrergic neurons^27^, and that these defects could contribute to intestinal dysmotility that persists even after the surgery in many HSCR patients. In the current study, we determined the expression and distribution of Sema3A and its receptor NRP1 in the rat developing gut and studied the role of Sema3A on neuronal connectivity. Our data showed that Sema3A restricts axon extension and synapse formation of enteric neurons. Comparison of the ganglionic colon of HSCR patients to the colon of patients with anorectal malformation showed reduced expression of the synaptic molecule synapsin 1 in HSCR. Interestingly, synapsin 1 expression in HSCR was inversely correlated with Sema3A expression level. Our study identifies Sema3A as a critical regulator of ENS connectivity and provides a putative link between altered ENS connectivity and HSCR.

## Results

### Expression and distribution of Sema3A and NRP1 in rat distal colon

**Figure 1:**
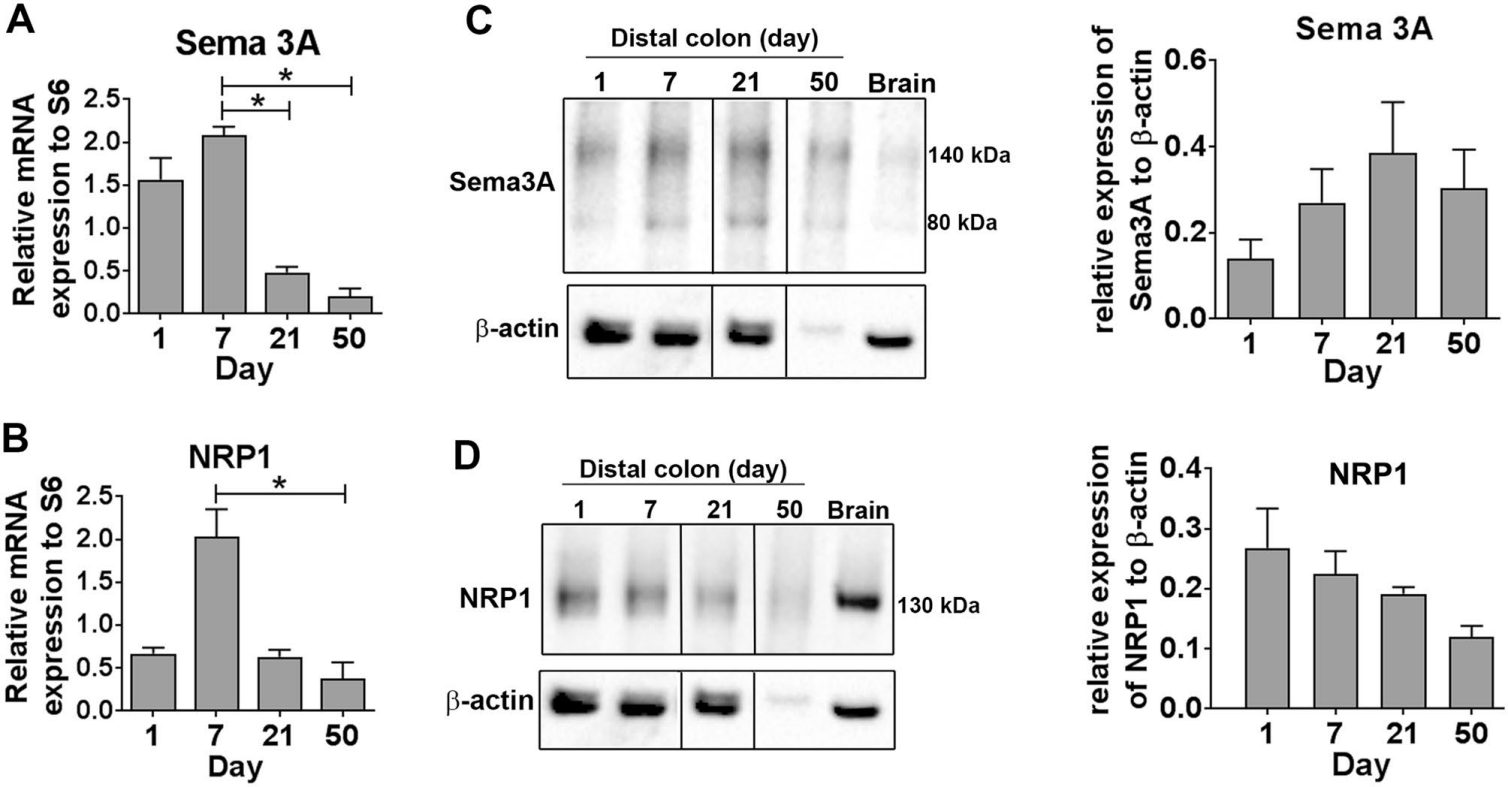
The expression of Sema3A and NRP1 is developmentally regulated in the rat colon. (**A**,**B**) PCR amplification of Sema3A (**A**) and NRP1 (**B**) mRNA extracted from rat colon at 1, 7, 21 and 50 days. Expression levels are normalized to the ribosomal S6 gene. Data represent means ± SEM (n = 5). *p < 0.05, Kruskal–Wallis test followed by Dunn’s post-test. (**C**,**D**) Western blot of distal colon lysates prepared from rats at 1, 7, 21 and 50 days and of adult rat brain as positive control. For each sample, 10 µg of proteins were loaded on a 4–12% LDS-NuPage and immunoblotted with antibodies against Sema3A (**C**) or NRP1 (**D**) and β-actin. The dividing lines in (**C**,**D**) delineate the position of the cropping performed on the same gel before grouping. The full-length blots are presented in Supplementary Information S1 and S2. The quantification of the Sema3A and NRP1 signal intensity was normalized to β-actin signal in the same sample. Western blot for Sema3A and NRP1 were performed on the same nitrocellulose membrane and share therefore the same β-actin membrane. Data represent means ± SEM (n = 3–5).

To examine whether Sema3A and its obligatory receptor NRP1 were expressed in the developing rat gut, mRNA and protein expression were assessed in rat distal colon on days 1, 7, 21 and 50 by qPCR and Western blot respectively. Expression of Sema3A and NRP1 mRNA was detected at all ages and showed higher expression levels during the early postnatal period, with a peak at day 7 for both Sema3A and NRP1 (Fig. 1A,B). For Western blot analysis, we used a Sema3A antibody for which we first assessed the specificity toward Sema3A in transfected COS-7 cells. The Sema3A antibody yielded a major band at 90 kDa as well as a faint band at 80 kDa in COS-7 cells transfected with myc-Sema3A cDNA while no signal was apparent in COS-7 cells transfected with a control vector encoding eGFP (Supplemental Information S4). Western blot of rat distal colon for Sema3A yielded two main bands of ~ 140 and ~ 80 kDa in gut and brain samples (Fig. 1C). Quantification indicated no statistical difference for Sema3A protein expression over time between 1 and 50-day old (Fig. 1C). In parallel, NRP1 protein expression also showed no variation over time (Fig. 1D).

**Figure 2:**
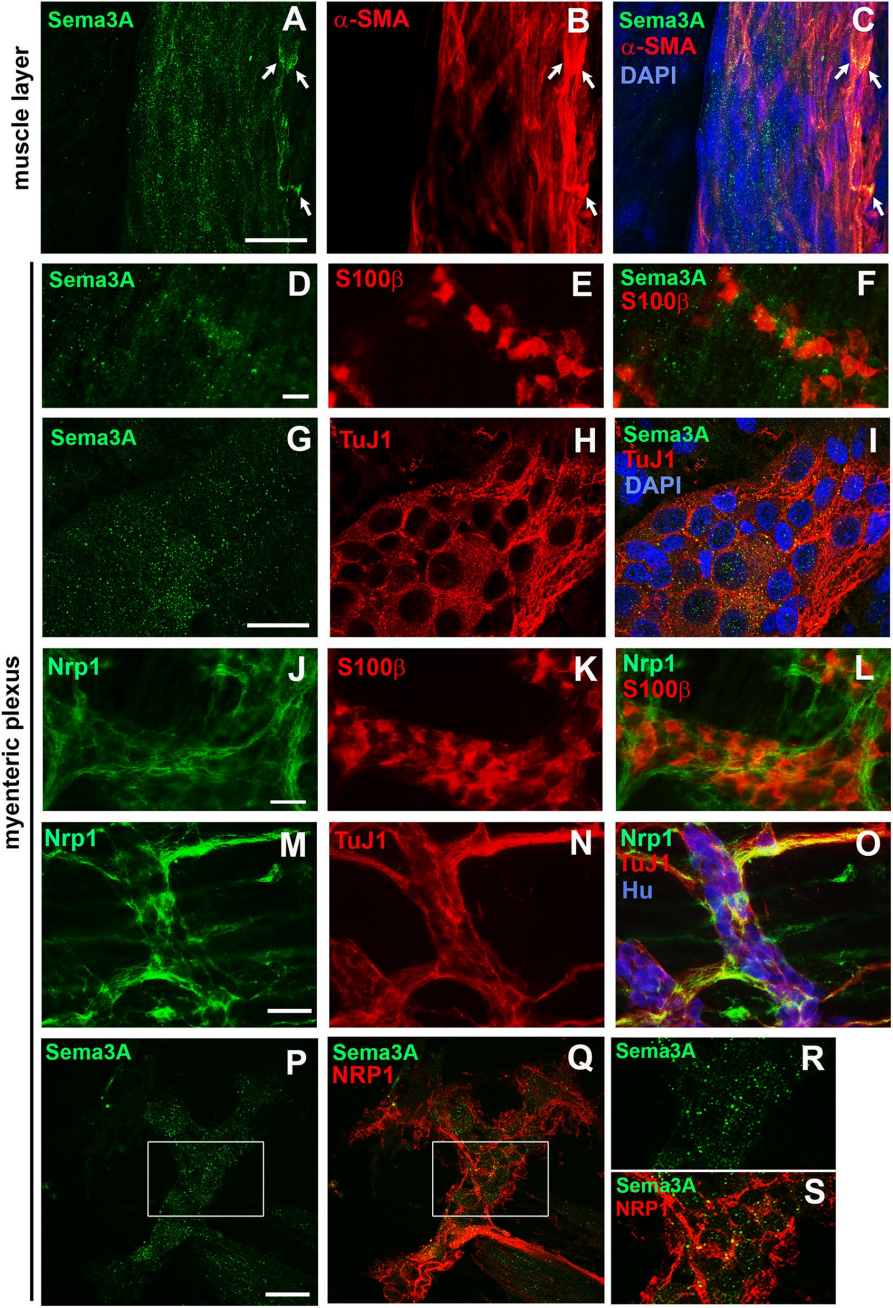
Sema3A and NRP1 are both associated with ganglia in the myenteric plexus of the distal colon in 7- and 36-day-old rats. (**A**–**C**) Double immunolabeling for Sema3A (**A**,**C** in green) and α-SMA (marker for muscle cells; **B**,**C** in red) in the muscle layer of 7-day-old rat distal colon. Cell nuclei are labeled with DAPI. Sema3A immunostaining appears as numerous small puncta throughout the tissue, closed to muscle cells. In some instances, a higher concentration of Sema3A labeling, more uniformly distributed, is associated with muscle cells (arrows). (**D**–**F**) Double immunolabeling for Sema3A (**D**,**F** in green) and S100β (marker for enteric glial cells; **E**,**F** in red) in the myenteric plexus of 7-day-old rat distal colon. (**G**–**I**) Double immunolabeling for Sema3A (**G**,**I** in green) and TuJ1 (marker for neuronal soma and axonal processes; **H**,**I** in red) in the myenteric plexus of 7-day-old rat distal colon. Cell nuclei are labeled with DAPI. (**J**–**L**) Double immunolabeling for NRP1 (**J**,**L** in green) and S100β (**K**,**L** in red) in the myenteric plexus of 7-day-old rat distal colon. (**M**–**O**) Triple immunolabeling for NRP1 (**M**,**O** in green), TuJ1 (**N**,**O** in red) and Hu (marker for neuronal soma, **O** in blue) in the myenteric plexus of 7-day-old rat distal colon. (**P**,**Q**) Double immunolabeling for Sema3A (**P**, in green) and NRP1 (**Q**, in red) in the myenteric plexus of 36-day-old rat distal colon. (**R**,**S**) Higher magnification of the boxed regions in (**P**,**Q**). Scale bars **A**–**C**,**G**–**I** 15 µm; **D**–**F**,**J**–**Q** 50 µm.

To characterize the tissue distribution of Sema3A and NRP1 proteins more specifically within the ENS, whole mount preparations of 7-day-old rat myenteric plexus from distal colon were immunostained for Sema3A or NRP1 and with markers of muscle cells (αSMA), neurons (Hu, and TuJ1) and glial cells (S100β). Within the muscle layer, Sema3A immunoreactivity exhibited a punctate staining but also, in a few instances, a more homogeneous profile in muscle cells (Fig. 2A–C). Sema3A immunoreactivity was also found at the interface between the muscle layer and the myenteric plexus. In addition, Sema3A immunoreactivity was observed within the myenteric plexus itself in which Sema3A puncta were predominantly found within ganglia throughout the axonal network but not particularly concentrated in the cytoplasm of enteric neurons or glia (Fig. 2D–I). Confocal microscopy serial optical sections of the tissue along the z-axis revealed that the Sema3A puncta were the brightest and the most numerous at the interface between the muscle layer and the myenteric plexus and within myenteric ganglia. NRP1 immunoreactivity was detected in muscle cells, endothelial cells and neurons, but not in enteric glial cells (Fig. 2J–L). In the myenteric plexus, NRP1 was mostly associated with neurons, particularly with axonal fibers (Fig. 2M–O). The same distribution for Sema3A and NRP1 was found in the distal colon of 7- and 36-day-old rats. Within the myenteric plexus, we observed a close proximity of Sema3A and NRP1 labeling, both restricted to the ganglia. This suggests that Sema3A proteins are localized in a position to interact with neuronal NRP1 in the myenteric plexus (Fig. 2P–S).

### NRP1 is enriched at growth areas of developing axons

**Figure 3:**
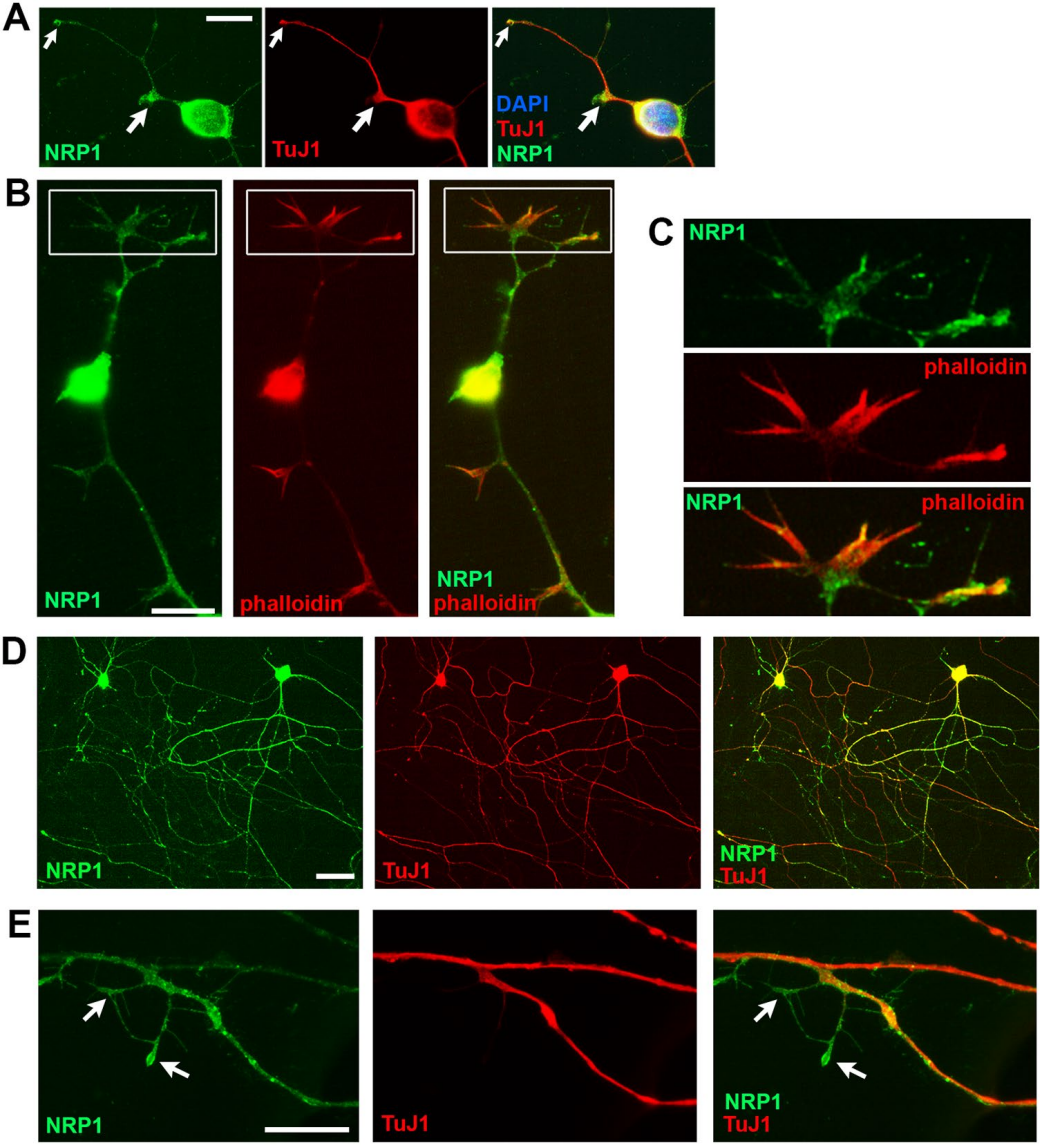
NRP1 is enriched at growth areas of developing enteric neurons in rat primary cultures (**A**) double immunolabeling for NRP1 (green) and TuJ1 (red) at 1 day of culture. NRP1 is enriched at the tips (small arrow) and buds (large arrow) of the developing axons. Cell nuclei are labeled with DAPI. (**B**,**C**) Double labeling of NRP1 (green) with Alexa Fluor 568-phalloidin (red) at 1 day of culture shows expression of NRP1 at growth cones. (**C**) Higher magnification of the boxed regions in (**B**). (**D**,**E**) Double immunolabeling for NRP1 (green) and TuJ1 (red) at 7 days of culture. NRP1 is widely localized in the profuse network of TuJ1-immunoreactive axons as well as with thin extending axonal ramifications (arrows in **E**). Scale bars (**A**,**B**,**E**) 12 µm; (**D**) 30 µm.

To better characterize NRP1 distribution and localization in developing neurons, we performed primary culture of rat ENS processed for NRP1 and TuJ1 immunostaining at different developmental stages. At 1 day of culture, a stage corresponding to the emergence of the axons, NRP1 was observed at the periphery of the neuronal cell body as well as on axons, with a particularly high concentration at the tips and buds of developing axons and emerging branching points (Fig. 3A). Double labeling of NRP1 with phalloidin, which binds F-actin that is highly enriched at growth cones, indicated that NRP1 was localized at neuronal growth cones (Fig. 3B,C). At 7 days of culture, corresponding to a stage of gradual increase of axonal complexity, NRP1 was detected in neuronal cell body and was widely associated with the profuse axonal network (Fig. 3D). As seen at 1 day of culture, thin extending secondary axonal fibers expressed NRP1 proteins with a striking accumulation at the tip of the processes (Fig. 3E). These results suggest that NRP1 receptors localize at specific neuronal subdomains important for axonal development and patterning.

### Sema3A inhibits axonal outgrowth of enteric neurons

**Figure 4:**
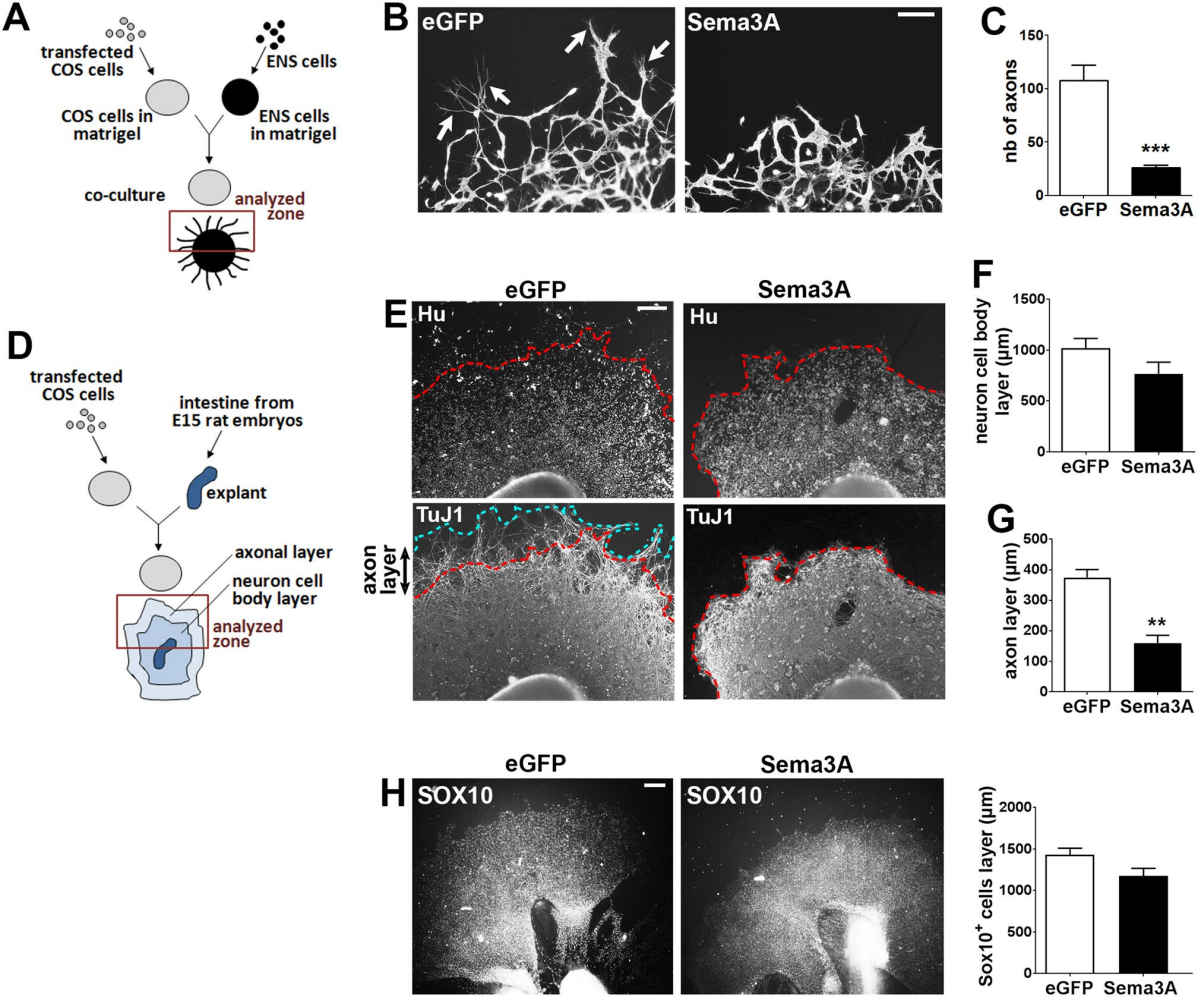
Sema3A inhibits axonal outgrowth in dissociated cell cultures and gut explants. (**A**) Schematic illustrating the coculture of enteric neurons with transfected COS-7 cells in matrigel. (**B**) Enteric neurons aggregated in matrigel were co-cultured with eGFP- or Sema3A-transfected COS-7 cells and were immunolabeled for TuJ1. In cocultures with eGFP-transfected COS-7 cells, numerous axonal fibers extend out of the matrigel dot (arrows). Scale bar 100 µm. (**C**) Quantification of the number of axons extending outside the matrigel dot. Data represent means ± SEM (n = 8 from 4 independent experiments); ***p = 0.0002, Mann–Whitney test. (**D**) Schematic illustrating the coculture of gut explants with transfected COS-7 cells in matrigel. (**E**) Gut explants co-cultured with eGFP- or Sema3A-transfected COS-7 cells were immunolabeled for Hu and TuJ1. The dotted red line corresponds to the enteric neurons migration wavefront from the explant. The dotted blue line corresponds to the border of axonal projections. Scale bar 250 µm. (**F**) Quantification of the thickness of the neuronal cell body layer. (**G**) Quantification of the thickness of the axonal layer. Data represent means ± SEM (n = 7 from 3 independent experiments); **p = 0.0012, Mann–Whitney test. (**H**) Gut explants co-cultured with eGFP- or Sema3A-transfected COS-7 cells were immunolabeled for SOX10 to assess the migration of precursor and glial cells out of the explants. Data represent means ± SEM (n = 7).

**Figure 5:**
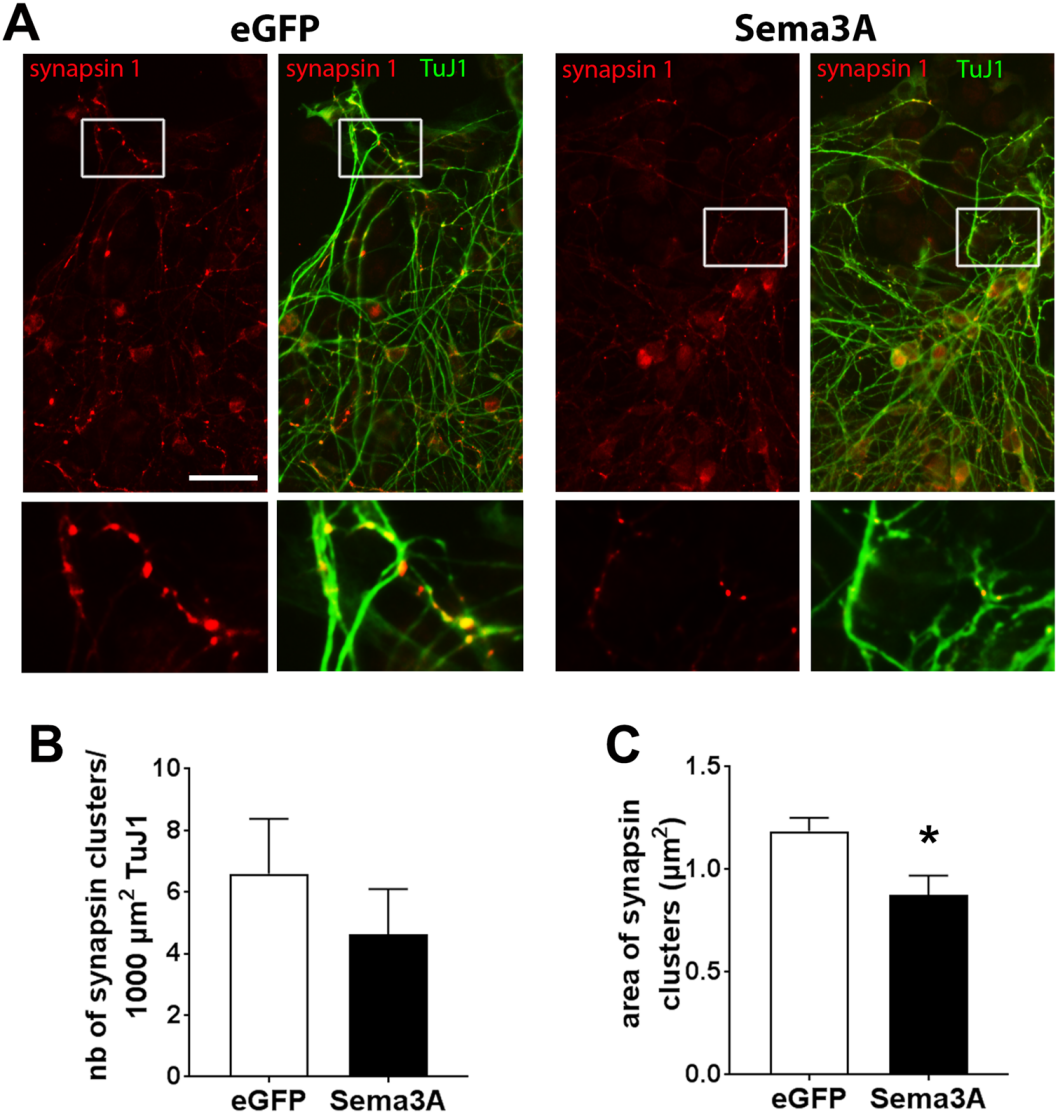
Sema3A restrains the size of synapses. (**A**) Gut explants cocultured with eGFP- or Sema3A-expressing COS-7 cells were immunolabeled with synapsin 1 and TuJ1 antibodies. Images at the bottom represent higher magnification of the boxed region. Scale bar 35 µm. (**B**,**C**) Quantification of the number of synapsin 1 clusters per 100 µm^2^ of Tuj1 labeling (**B**) and area of synapsin 1 clusters (**C**). Data are mean ± SEM (n = 5); *p = 0.032, Mann–Whitney test.

Based on our finding that NRP1 receptors localized at appropriate positions to regulate axonal outgrowth, we examined whether Sema3A could affect axon development of enteric neurons. Dissociated gut cells prepared from E15 rat embryos previously resuspended in matrigel were cocultured with Sema3A-transfected COS-7 cells placed in matrigel (Fig. 4A). For control condition, we used COS-7 cells transfected with a vector encoding eGFP. The number of axons from enteric neurons that extended outside the matrigel dot was counted in the area facing the COS-7 cells. We found that Sema3A induced a dramatic decrease of the number of axons detected outside of the matrigel dot (Fig. 4B,C). The effects of Sema3A on axonal outgrowth were also studied on gut explants, a model that preserves the integrity of the tissue (Fig. 4D). Gut explants cultured with eGFP-transfected COS-7 cells showed an extensive colonization of enteric neurons with profuse axonal extensions all around the explants (Fig. 4E). When gut explants were cocultured with Sema3A-expressing COS-7 cells, the layer of enteric neurons was similarly established, but the extent of the axonal arbor was greatly reduced (Fig. 4E). The thickness of the layer containing the neuron cell bodies indicated similar values for explants cultured in the presence of eGFP- or Sema3A-transfected COS-7 cells (Fig. 4F). By contrast, the thickness of the axonal layer was decreased in the presence of Sema3A-transfected COS-7 cells as compared to eGFP-transfected cells (159 ± 25 µm vs. 374 ± 25 µm respectively; n = 7; p = 0.001 Mann–Whitney test; Fig. 4G). Next, we assessed whether neural precursor or glial cells were affected by Sema3A stimulation by measuring the distance of migration of SOX10-immunoreactive cells. As shown in Fig. 4H, the thickness of the SOX10-containing layer was similar for gut explants cultured with eGFP- or Sema3A-expressing COS-7 cells. To further study the effects of Sema3A on neuronal maturation, the number and size of synapses within the axonal network were analyzed in gut explants immunolabeled for synapsin 1, a presynaptic protein associated with synaptic vesicles previously shown to be a robust general marker of synapses^28^. Gut explants grown with Sema3A-expressing COS-7 cells showed no change in the number of synapses but a decrease in the area of synaptic clusters compared to explants grown with eGFP-expressing COS-7 cells (Fig. 5A–C; 0.87 ± 0.09 vs. 1.19 ± 0.06 µm^2^ respectively; p = 0.032 Mann–Whitney test). Previous studies conducted in peripheral cells reported that Sema3A stimulation induced a cellular redistribution of NRP1 characterized by receptor internalization in intracellular vesicles^29^. We thus analyzed in gut explants whether NRP1 distribution was modified upon Sema3A stimulation. No difference in NRP1 distribution was noticed between gut explants cultured with eGFP- and Sema3A-expressing COS-7 cells (Supplemental Information S6). Altogether, these results suggest that Sema3A restrains axon extension and synaptic connectivity of enteric neurons.

### HSCR is associated with reduced expression of the synaptic protein synapsin 1

**Figure 6:**
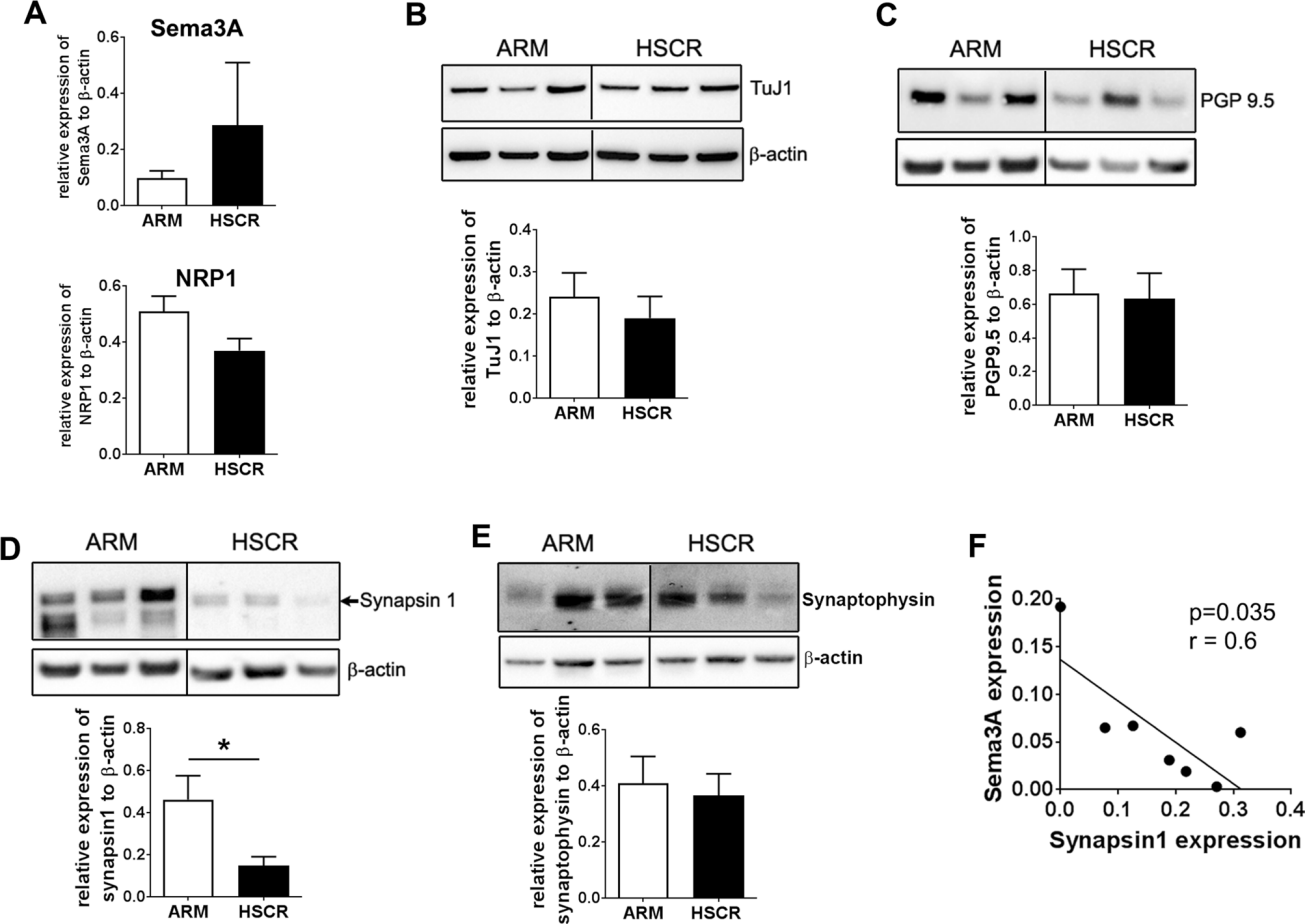
The reduced level of synapsin 1 in the ganglionic colon of HSCR is inversely correlated with Sema3A expression. (**A**) Quantification by Western blot of Sema3A and NRP1 protein level normalized to β-actin. Data represent means ± SEM (n = 8–9). (**B**–**D**) Western blot of the colon of three representative ARM and HSCR patients with the corresponding quantification for TuJ1 (**B**), PGP9.5 (**C**), synapsin 1 (**D**) and synaptophysin (**E**). The dividing lines in the Western blot in (**B**–**E**) delineate the position of the cropping performed on the same gel before grouping. The full-length blots are presented in Supplementary Information S3. Data represent means ± SEM (n = 8–9). *p = 0.035, Mann–Whitney test. (**F**) Linear regression analysis between Sema3A and synapsin 1 expression in HSCR colon; p = 0.035, Spearman’s test.

To begin to address the pathophysiological implications of Sema3A/NRP1 expression in ENS developmental disorders, we assessed the protein expression of Sema3A and its receptor NRP1 in the ganglionic sigmoid colon of HSCR patients, considered by pathologists as the healthy segment, compared to the sigmoid colon of a group of patients without HSCR but with anocolorectal malformation (ARM). Although Western blot analyses indicated an average value for Sema3A in HSCR samples three times higher than for ARM patients, no significant difference was observed either for Sema3A or NRP1 in HSCR compared to ARM patients (Fig. 6A). To explore whether the ENS neuronal network might be altered in HSCR patients, the protein expression of the neuronal markers TuJ1 and PGP9.5 were studied by Western blot. No difference was observed between the two groups for TuJ1 and PGP9.5 (Fig. 6B,C). To examine whether synapses might be altered, we analyzed the expression of synapsin 1 and synaptophysin, two synaptic proteins previously shown to be the most robust and reliable general markers to label synapses^28^. A decreased expression of synapsin 1 was found in HSCR compared to ARM patients while no difference was observed for synaptophysin between the two groups of patients (Fig. 6D,E). Interestingly, a significant negative correlation was observed between the expression of Sema3A and synapsin 1 in HSCR patients (Fig. 6F).

## Discussion

Uncovering the mechanisms involved in the formation of enteric neuronal circuitry is critical to better understand the pathophysiology of gut dysfunctions, and in particular motility disorders. In this study, we provide evidence that Sema3A and its receptor NRP1 are expressed in the developing gut and that Sema3A exerts a negative control on axonal elongation and synaptic connectivity during ENS maturation. In addition, ganglionated bowel segment from HSCR patients, considered by pathologists as the healthy segment, showed a reduced expression of the synaptic molecule synapsin 1, which was inversely correlated with Sema3A expression levels. Our study provides proof of a novel role of Sema3A in regulating enteric neuronal connectivity and suggests there may be ENS synaptic defects in the normoganglionic colon of HSCR that could contribute, in part, to post-operative complications in HSCR patients.

Our study demonstrated that Sema3A and its receptor NRP1 are expressed in the rat gut at early postnatal stages. At the protein level, we observed two major bands for Sema3A at 140 and 80 kD. The expected molecular weight of Sema3A deduced from its amino acid sequence is 90 kDa, which corresponds to the size that we observed for Sema3A expressed in COS-7 cells. The 140 kDa form found in rat colon likely results from post-translational modifications, such as glycosylations, of the 90 kDa form. The 140 kDa form of Sema3A in the colon is consistent with the reported molecular weight ranging from 95 to 160 kDa in brain tissue^30^. On the other hand, the 80 kDa band detected in both the colon and COS-7 cells likely results from proteolytic processing as furin-dependent cleavage of Sema3A has been shown to occur in mammalian cells^31^. Analyses of mRNA and protein expression levels were performed on whole colon tissues and therefore cannot document on the specific cell type expressing these molecules. However using immunostainings of dissected colonic myenteric plexus we were able to provide evidence that Sema3A and NRP1 were expressed in myenteric neurons and/or muscle cells for Sema3A. Indeed, Sema3A harbored a punctate pattern located in between the muscle cells layer and the myenteric plexus as well as within myenteric ganglia. The punctate pattern for Sema3A observed in the gut is similar to the one described in previous studies carried out on brain slices or in cultures of central neurons^32–34^. Our own data from transfected COS-7 cells also showed an extracellular punctate staining with Sema3A-expressing COS-7 cells while such pattern was not observed with eGFP, a non-secreted protein (Supplemental Information S5). The spread clustered distribution of Sema3A in brain tissues and neuron cultures has been attributed to the presence of secreted Sema3A linked to the proteoglycans of the extracellular matrix^32–34^. Similarly, Sema3A puncta detected in the muscle cell layer and in the myenteric plexus could correspond to secreted Sema3A trapped in the extracellular matrix. The cellular source of Sema3A in the gut remains incompletely characterized and could involve muscle and ENS cells. In situ hybridization studies for Sema3A mRNA performed in E14.5 mouse embryo and in 3-day-old mice indicated that Sema3A-synthetizing cells were detected within the inner mesenchyme, probably associated with the longitudinal muscle cell layer, but absent from the ENS^23,24^. Accordingly, we observed in our study that a few muscle cells exhibited a higher concentration of Sema3A labeling with a more uniform pattern, a distribution consistent with a Sema3A biosynthesis by these cells but further investigation would be required to identify the source of Sema3A in the myenteric plexus. We found that the receptor NRP1 was associated with enteric neurons, in particular with their axonal arbor. Altogether, the cellular distribution of Sema3A and NRP1 in the rat distal colon suggests that Sema3A produced and secreted by muscle or ENS cells would target the axons of NRP1-expressing enteric neurons.

The tissue expression and distribution of Sema3A and NRP1 observed in this study during the early postnatal period supports the possibility that Sema3A/NRP1 signaling might contribute to the shaping of neuronal circuitry during this period. Indeed, major changes in ENS structure occur during the early postnatal period, including gangliogenesis, axonal outgrowth, patterning of axonal fibers in interganglionic bundles and synapse formation^10–14^. These changes in neuronal morphology and connectivity are thought to be controlled by a combination of stimulatory and inhibitory signals resulting ultimately in the proper patterning of the enteric neuronal network. Using in vitro and ex vivo models of developing ENS, we found that Sema3A reduced axonal outgrowth and the size of synaptic clusters of enteric neurons suggesting that Sema3A is part of the inhibitory molecular repertoire involved in the shaping of neuronal connectivity during the postnatal period. Most of the effects induced by Sema3A have been shown in previous studies to be mediated via a receptor complex that contains NRP1, as the ligand binding subunit, and one of the four Plexin A coreceptors, as the signal-transducing subunit^35^. Due to its ligand binding function, NRP1 is considered to be an obligatory receptor for the transduction of Sema3A-induced signaling. Our findings showed that NRP1 was strikingly present at growth areas of developing axons, such as neurite growth cones and buds as well as thin axonal protrusions, supporting the hypothesis that Sema3A regulates axon development in enteric neurons through interaction with NRP1. The inhibitory activity of Sema3A on axonal elongation and synaptic connectivity of enteric neurons is in agreement with previous studies reported in the central nervous system showing that several members of the class 3 semaphorins inhibit axon extension and synapse formation of hippocampal neurons, leading to modulation of synaptic transmission^36–39^. In the peripheral nervous system, Sema3A has been shown to control the growth and navigation of spinal motor neuron axons to the muscle targets in the forelimb, therefore underlying the establishment of functional motor circuitry^40^. The Sema3A-induced regulation of enteric neuronal connectivity identified in the present study could have major consequences on neurally-dependent gut functions, such as gastrointestinal motility. Indeed, it has been shown that anomalies in the ENS axonal tract configuration could result in severe bowel dysmotility^9^ and that the complexity of neuronal connectivity correlated with intestinal motility pattern^41^. Whether Sema3A, by regulating enteric neuronal network, could control gut contractile activity remains to be demonstrated.

Previous studies pointed out a link between Sema3A and HSCR as deleterious mutations of Sema3A have been reported in some HSCR patients^24,25^. Furthermore, increased Sema3A protein expression have been described in the endothelin receptor-B null mouse model of HSCR and in the colon of some HSCR patients^25,26,42^. In our pilot study, despite an average value of Sema3A signal intensity three times higher in Western blot for HSCR than ARM patients, we did not find any statistical difference in Sema3A protein level between the two groups. This might result from the limited number of patients included in this study and from a large heterogeneity of Sema3A protein level in the HSCR group since variations up to 20-fold have been observed between individuals of this group. The variation of Sema3A expression might also reflect the age distribution HSCR patients and the reported genetic and phenotypic heterogeneity of this population^43^. These results might therefore be confirmed in the future on a larger cohort of patients. Regarding the expression of neuronal molecules, we found a marked reduction of the synaptic marker synapsin 1, but not that of synaptophysin, in the colon of HSCR compared to ARM patients, while the pan-neuronal markers TuJ1 and PGP9.5 were unaffected. This result suggests that the main neuronal processes and synapses are preserved, but that defects in specific synaptic proteins occur in HSCR. Synapsin 1 is a neuronal protein concentrated in synaptic vesicles that are present in nerve terminals. The role of synapsin 1, mainly studied so far in the central nervous system, is linked to several key neuronal development stages and functions, such as axonal elongation, formation of presynaptic terminals, regulation of the synaptic vesicle docking and synaptic transmission^44–46^. Our results showing a decreased level of synapsin 1 in HSCR compared to ARM patients suggest that defects in synapse formation or synaptic transmission between neurons or with gut target cells might occur in HSCR. Previous studies have also reported ENS abnormalities in the ganglionic segment of HSCR patients, such as the overexpression of polysialylated neural cell adhesion molecules, used as a marker of neuronal immaturity^47^. These data, together with our present results, suggest that impaired ENS maturation and connectivity might occur in the normoganglionic gut of HSCR which could contribute to altered gastrointestinal functions, such as motility disturbance often reported in HSCR patients, even after surgery.

Interestingly, we found a negative correlation between Sema3A and synapsin 1 protein expression level in the colon of HSCR patients. In view of our results showing an inhibitory activity of Sema3A on axonal outgrowth and on the size of synaptic clusters, one hypothesis is that the level of Sema3A expression might control the wiring of neuronal circuitry and synaptic connections. So far, the functional consequences of altered Sema3A expression on gut physiology are largely unknown but we already know that the lack of Sema3A in fetal mice induces a premature entry of sacral neural crest-derived cells into the hindgut^23^. However, knockdown of Sema3A during Zebrafish embryogenesis has no effect on the number of enteric neurons in the gut at embryonic stage^24^. Whether impaired Sema3A-activated signaling during the postnatal period induces defects in the maturation of neuronal connectivity remains to be determined. However, based on our results, one could envisage that defects in Sema3A signaling might impact the pattern of axonal projection and neuronal connections in between neurons and with gut target cells established during ENS postnatal development.

In conclusion, our study provides proof of a novel role of Sema3A in regulating enteric neuronal connectivity and uncovered possible ENS synaptic defects in the normoganglionic colon in HSCR that could contribute, in part, to post-operative complications in HSCR patients.

## Methods

### Study approval

For study involving rat samples, all experimental protocols were carried out in accordance with the relevant guidelines and regulations after obtaining the approval by the French Ministry of Research (Agreement # 02476.03). Studies involving human samples were performed in accordance with ethical guidelines and recommendations of the Declaration of Helsinki and received IRB approval by Nantes Ethical Committee under protocol number 12-16-2014. The database is in compliance with the requirements of the French data protection authority (CNIL, 08/07/2015-no. 915187) and registered by the French Ministry of Research under protocol 03/05/2015-no. 15.071bis. The EnteHirsch biobank was approved by the Clinical Ethics Committee (CPP Ouest IV of Nantes, 11/09/2014-no. DC-2011-1399). Parents or the legal guardians gave a written informed consent prior to inclusion in the study.

### Human colon tissues

Nine HSCR patients (6 boys), with a short-segment HSCR limited to the sigmoid colon, born after 37 weeks of gestation and aged between 17 and 222 days of life at the time of curative surgery were included in pediatric surgery departments from eight French hospitals: Nantes, Brest, Poitiers, Angers, Le Mans, Tours, Armand Trousseau and Necker (Paris). The specimens were collected at the time of surgery from the ganglionated zone (confirmed by double histopathological reading), always at a similar area of the sigmoid colon for all HSCR patients. Only one dedicated pediatric surgeon (AD) was in charge of collecting the specimens from the resected segment with always the same pre-established protocol. Non-inclusion criteria were: preoperative enterocolitis, anastomosis in the transition zone, another intestinal malformation associated with gastrointestinal dysmotility and severe associated malformation. Nine patients (6 boys) with a diagnosis of anocolorectal malformation (ARM) requiring a colostomy creation or closure before 1 year of age (between 35 and 287 days of life) and born after 37 weeks of gestation were included and considered in our study as a comparison group. ARM is a congenital colorectal malformation related to abnormal anal sphincter and pelvic muscular complex inherent to this developmental disorder, but probably unrelated to an underlying gastrointestinal motility or ENS dysfunction. In our study, the males presented a high ARM with recto-bulbar or recto-prostatic fistula and the females presented an “intermediate” ARM with recto-vestibular fistula. The specimens were collected from the sigmoid colon after colostomy.

Specimens collected from HSCR and ARM patients were placed immediately in sterile Hank’s balanced salt solution (HBSS) kept at 4 °C on ice. Mucosa and submucosa were separated from the muscular tissue by microdissection. Samples were placed immediately in liquid nitrogen and stored at − 80 °C.

### Real-time quantitative PCR

Total RNA was extracted from the distal colon of 1-, 7-, 21- and 50-day-old rats using Nucleospin RNA II (Macherey Nagel, Hoerd, France) and the resulting RNAs were reverse-transcribed using SuperScript III Reverse Transcriptase (Life Technologies, Saint-Aubin, France). Real-time quantitative PCR (qPCR) was performed using a StepOne thermocycler with a FastSybr green mastermix kit (Applied Biosystems, Foster City, CA, USA). The sense and anti-sense oligonucleotide primers used were as follows: rat Sema3A sens: 5′-TACTGCAAAGAGGCGCACAA-3′, antisens: 3′-GGCTCTCTGTGACTTCGGAC-5′; rat NRP1 sens: 5′-CAGCGATAAATGTGGCGGGA-3′, antisens: 3′-GTCATACTTGCAGTCTCTGTCCTC-5′. Internal controls were generated by amplifying the ribosomal protein S6 gene (S6) mRNA. The relative expression of the gene of interest was measured by the 2-∆∆Ct method. Analyses were performed on 5 animals for each studied age.

### Western blot

Distal colon from 1-, 7-, 21- and 50-day-old rats and resected colon from HSCR and ARM patients were lysed in a solution containing 50 mM Tris, 100 mM NaCl, 1% Triton X-100, 1 mM EGTA and protease inhibitors (Complete; Roche, France), pH 7.4, with a tissue homogenizer (Precellys 24, Bertin Technologies, France) and sonicated three times for 5 s. For brain preparation, forebrains of 50-day-old rats were homogenized in RIPA lysis buffer (50 mM Tris, 150 mM NaCl, 1% NP40, 0.5% sodium deoxycholate, 0.1% SDS, 1 mM EDTA, pH8.0) and centrifuged at 20,000*g* for 20 min to collect the supernatant. Equal amounts of lysate (10 μg of proteins) were separated using the Invitrogen NuPage Novex 4–12% Bis-Tris MidiGels^®^ and blotted onto a nitrocellulose membrane. Membranes were incubated overnight with a rabbit anti-Sema3A antibody (Santa Cruz, 1/500 for rat samples; 1/300 for human samples) or a goat anti-NRP1 antibody (R&D System, 1/2000 for rat samples; 1/500 for human samples), a mouse anti- class III β-tubulin (TuJ1; Abcam, 1/1,000), a rabbit anti-PGP9.5 (Invitrogen, 1/400), a rabbit anti-synapsin 1 (Sigma, 1/1,000), a mouse anti-synaptophysin (Cell Signaling, 1/200) and a mouse anti β-actin antibody, (Sigma, 1/5,000). Proteins were detected by the appropriate HRP-conjugated secondary antibodies (Thermoscientific, 1/5,000) using chemiluminescence (Clarity Western ECL Substrate, Bio-Rad).

### Immunofluorescence staining

#### Tissue

Segments of distal colon from 7- and 36-day-old rats were fixed in 0.1 M phosphate-buffered saline (PBS) containing 4% paraformaldehyde (PFA) at room temperature (RT) for 3 h. Whole mounts of longitudinal muscle and myenteric plexus (LMMP) were obtained by microdissection and were permeabilized with PBS containing 10% BSA, 0.5% Triton X-100 and 0.01% sodium azide for 2 h at RT. Tissues were then incubated with the following primary antibodies: rabbit anti-Sema3A (Santa Cruz, 1/200), goat anti-NRP1 (R&D System, 1/33), mouse anti-α smooth muscle actin (αSMA, Abcam, 1/500), mouse or rabbit anti-TuJ1 (Sigma or Abcam respectively, 1/1,000), mouse anti-S100β (Abcam, 1/500) and mouse or rabbit anti-HuC/D (HU; Molecular Probes or Santa Cruz respectively, 1/500) diluted in PBS containing 3% BSA, 0.25% Triton X-100 and 0.01% sodium azide for 16 h at RT. After washing, tissues were incubated for 2 h at RT with the appropriate Alexa 488-, Cy3-, Cy5- or Alexa 647-conjugated secondary antibodies (Jackson ImmunoResearch), and then incubated for 5 min in DAPI (ThermoFisher Scientific, 1/5,000 and mounted with ProLong Gold Antifade Reagents (ThermoFisher Scientific).

#### Cell culture and gut explant

Cells or gut explants were fixed in PBS containing 4% PFA for 15 min, permeabilized for 5 min in PBS containing 0.25% TritonX-100 and incubated in PBS containing 10% BSA and 0.01% sodium azide for 30 min at RT. The cells were then incubated with the following primary antibodies diluted in PBS containing 3% BSA and 0.01% sodium azide: goat anti-NRP1 (1/500), mouse anti-TuJ1 (1/500), guinea-pig anti-synapsin 1 (Synaptic Systems, 1/700) or goat anti-SOX10 (Santa Cruz, 1/300) for 16 h at RT. For F-actin labeling, cells were incubated for 1 h at 37 °C with Alexa Fluor 568-phalloidin (1/10,000, Thermo Fisher). After washing, cells were incubated for 1h30 min at RT with the appropriate Alexa 488- or Cy3-conjugated secondary antibodies, and then for 5 min in DAPI 1/5,000 and mounted with ProLong Gold Antifade Reagents.

### ENS culture

#### Enteric neuron-glia coculture

To study the cellular distribution of NRP1 in enteric neurons during development, we used a model of neuron-glia coculture as previously described^11^. First, enteric glial cells were prepared from ENS culture derived from the intestine of E15 rat embryos^48^ and were plated at a density of 7,500 cells/cm^2^ in a 24-well plate in in DMEM containing 10% fetal calf serum (FCS), 2 mM glutamine and 50 μg/ml streptomycin and 50 UI/ml penicillin. After 4 days, the medium was replaced with serum-free Neurobasal/B27 medium (Gibco) 3 h before neuron culture. Rat enteric neuron culture was prepared from the intestine of E15 rat embryos. Briefly, intestine were removed, finely diced in Hank’s buffered salt solution and then triturated mechanically using a scalpel. Tissue fragments were incubated for 15 min with 0.25% trypsin (Invitrogen) and for 10 min with 0.1% DNase I (Sigma) and the dissociated cells were plated at 175,000 cells/cm^2^ on glass coverslips coated with poly-l-lysine (1 mg/ml, Sigma) in DMEM high glucose containing 10% FCS, 2 mM glutamine and antibiotics. The coverslips were then transferred 3 h later to the wells containing the enteric glia and 3 µM of 1-β-d-arabinofuranosylcytosine (Calbiochem) were added 24 h later. The cells were maintained for up to 7 days.

#### ENS culture in matrigel

To study the effects of Sema3A on enteric neuron development, dissociated cells from E15 rat embryos were prepared as described above and were resuspended in BD matrigel matrix (Corning #356234) at a concentration of 50,000 cells/μl, from which 3 μl were loaded in the center of a well of a 24-well plate previously coated with matrigel diluted 1/100 in Neurobasal medium. Three μl of transfected COS-7 cells resuspended in BD Matrigel Matrix Growth Factor Reduced (Corning #356230) (50,000 cells/μl) were then loaded 3 mm distant from the ENS matrigel dot. The ENS-COS-7 cells preparation in matrigel dots was cultured in Neurobasal/B27 medium containing antibiotics and 50 ng/ml GDNF for 4 days.

### Gut explants

The intestine of E15 rat embryos was dissected and cut into 6 equal pieces. Each piece was transfered to a well of a 24-well plate previously coated with 0.05 mg/ml collagen diluted in 20 mM acetic acid. The explants were cultured in DMEM high glucose (Gibco) containing 50 ng/ml GDNF, 10% FCS, 2 mM glutamine and antibiotics. After 1 day of culture, 3 μl of transfected COS-7 cells resuspended in BD Matrigel Matrix Growth Factor Reduced (50,000 cells/μl) were loaded 5 mm distant from the explant. The gut explant-COS-7 cells preparation was maintained for 3 more days.

### COS-7 cell culture and transfection

COS-7 cells were grown in DMEM containing 10% fetal calf serum (FCS) and antibiotics. Transfections with eGFP or Sema3A plasmids were performed using the Lipofectamine 2000 reagent (Invitrogen). To perform a COS-7 cell suspension in matrigel, the cells were gently scrapped one day after the transfection and homogenised in matrigel at a concentration of 50,000 cells/μl as described above.

### Equipment and settings

#### Western blots

The signals were detected using the Bio-Rad Gel-Doc imaging system and the quantifications were performed with Image J software. The values of Sema3A and NRP1 signal were normalized to the β-actin signal and expressed as a percentage of the average of controls. Image processing to adjust brightness and contrast were performed for the main figures presentation with Adobe photoshop software.

#### Image analysis and quantification

For whole-mount tissues, serial optical sections along the z-axis separated by 0.3 μm were acquired with a Nikon A1RSi confocal microscope, using appropriate laser wavelength and filters, with 20×/0.75 or 60×/1.4 oil immersion objectives. Images were processed with the Image J software (NIH, Bethesda, MD). Images of gut explants and ENS cultures were acquired with a digital camera (DP50, Olympus) coupled to a fluorescence microscope (BX51, Olympus) using 20× or 100× oil immersion objectives.

Images quantification was performed with the ImageJ software program. For ENS cultured in matrigel, the axonal outgrowth was measured by counting the number of axon extending out of the matrigel dot (Fig. 4A). Eight matrigel dots were analyzed from 4 independent experiments. For gut explants cultures, the wavefront of the enteric neurons migration and the border of axonal extension were traced according to the Hu and TuJ1 immunostaining respectively. The thickness of the neuron cell body layer corresponded to the distance between the explant border and the wavefront of neuron cell body migration. The thickness of the axonal layer corresponded to the distance between the enteric neurons migration wavefront and the border of axonal extensions (Fig. 4D). The thickness of the neuron cell body layer and of the axonal layer was measured in seven explants per conditions from 3 independent experiments. The distance of migration of precursor and glial cells out of the gut explants was measured between the border of the explant and the wavefront of SOX 10-immunoreactive cells. To determine the number and area of synapsin 1 clusters, images were acquired with InCell Analyzer 2200 microscope (GE Healthacre) using a 20× objective. The same exposure time was used for all conditions, thereby ensuring accurate comparisons. The number of synapsin clusters per surface unit of TuJ1 immunostaining was determined with ImageJ software in 4 randomly selected zones for each gut explants (n = 5). The fluorescence intensity threshold for clusters was defined as at least 2.5 times the average intensity of fluorescence in the underlying neuronal process as previously described^49^. Image processing to adjust brightness and contrast were performed for figure presentation with Adobe photoshop software.

### Statistics

Data analysis and statistics were performed using Excel and GraphPad Prism 5. Values indicated mean ± SEM. Group comparison was made by Mann–Whitney test or Kruskal–Wallis Test with Dunn’s post-hoc test as indicated. Correlations between variables were assessed by Spearman’s correlation coefficients, as appropriate. The level of statistical significance was set at *p < 0.05; **p < 0.01; ***p < 0.001.

## Supplementary information

Supplementary information
