# Supplementary material for: Semaphorin 3A controls enteric neuron connectivity and is inversely associated with synapsin 1 expression in Hirschsprung disease
Source: Sci Rep. 2020 Sep 15;10:15119. doi: 10.1038/s41598-020-71865-3 (PMC7492427; doi:10.1038/s41598-020-71865-3)

**Supplemental Information**

**Semaphorin 3A controls enteric neuron connectivity and is inversely associated with synapsin 1 expression in Hirschsprung disease**

Jacques Gonzales^1^, Catherine Le Berre-Scoul^1^, Anne Dariel^1^, Paul Bréhéret^1^, Michel Neunlist^1^ and Hélène Boudin^1^

^1^Université de Nantes, Inserm, TENS, The Enteric Nervous System in Gut and Brain Diseases, IMAD, Nantes, France

S1

Full-length Western blot for Sema3A in rat distal colon. The red boxes denote the regions used in the corresponding main figures.

­­_­­_
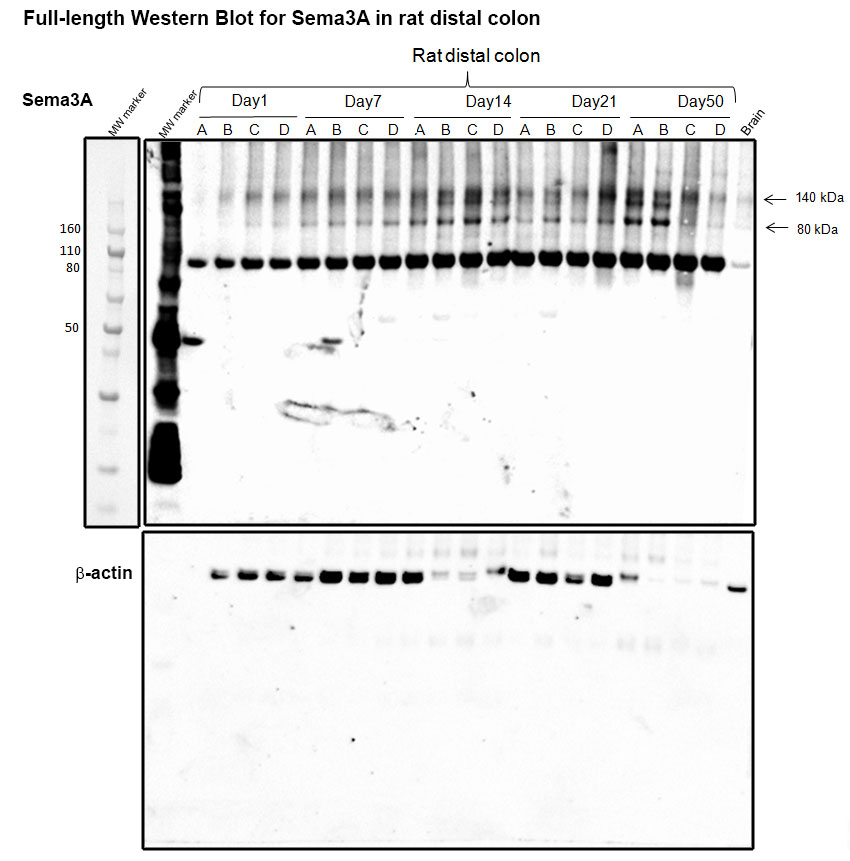


S2

Full-length Western blot for NRP1 in rat distal colon. The red boxes denote the regions used in the corresponding main figures. Note that NRP1 detection was performed on the same nitrocellulose membrane than Sema3A detection, and share therefore the same β-actin membrane.

­
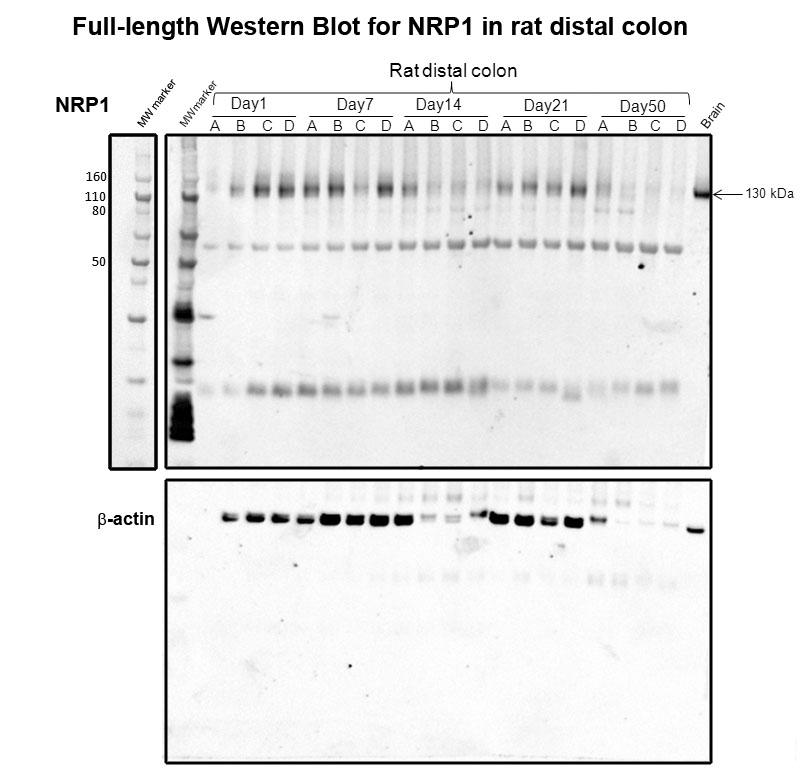


S3

Full-length Western blot for TuJ1, PGP 9.5, synapsin 1 and synaptophysin in colon from HSCR and ARM patients. Note that synapsin 1 and PGP9.5 detection were performed on the same nitrocellulose membrane, and share therefore the same β-actin membrane. The red boxes denote the regions used in the main figures. In the corresponding main figures, the ARM samples were presented on the left and the HSCR samples were presented on the right.


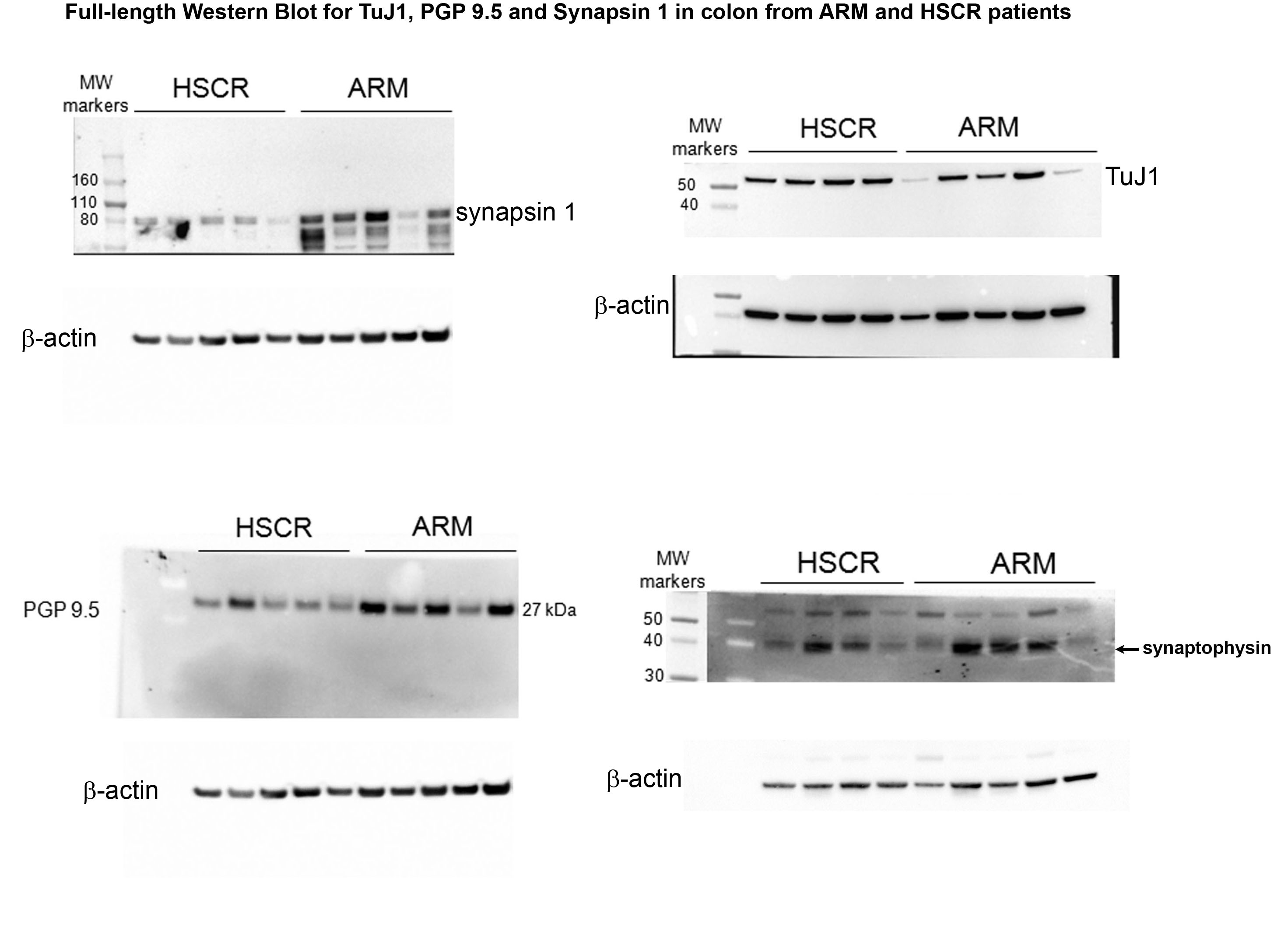


S4

Western blot of COS cells transfected with a control vector encoding eGFP or a myc-Sema3A cDNA revealed with anti-myc, anti-Sema3A and anti-β-actin antibodies.

_
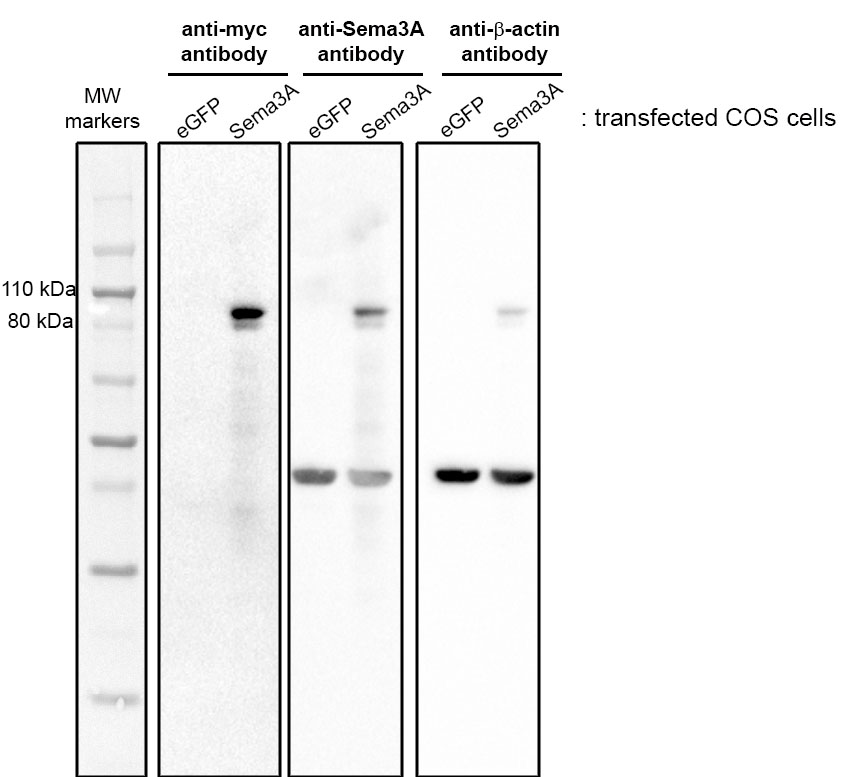
­­_

S5

Immunostaining of Sema3A on COS cells transfected with myc-Sema3A cDNA revealed a punctate staining surrounding the transfected COS cells. The negative control is COS cells transfected with eGFP cDNA encoding the protein eGFP, which is not secreted out of the cell.


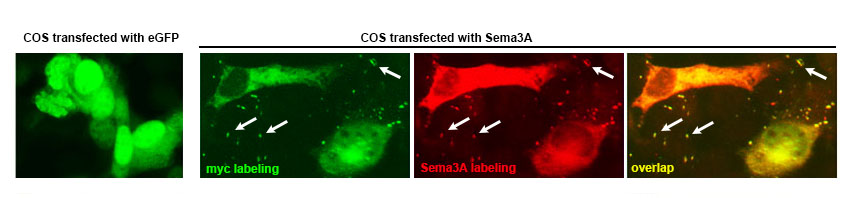


S6

Immunostaining for NRP1 and TuJ1 of rat gut explants cultured for 3 days with eGFP- or Sema3A-expressing COS cells. No major changes in NRP1 distribution was observed between the two conditions.


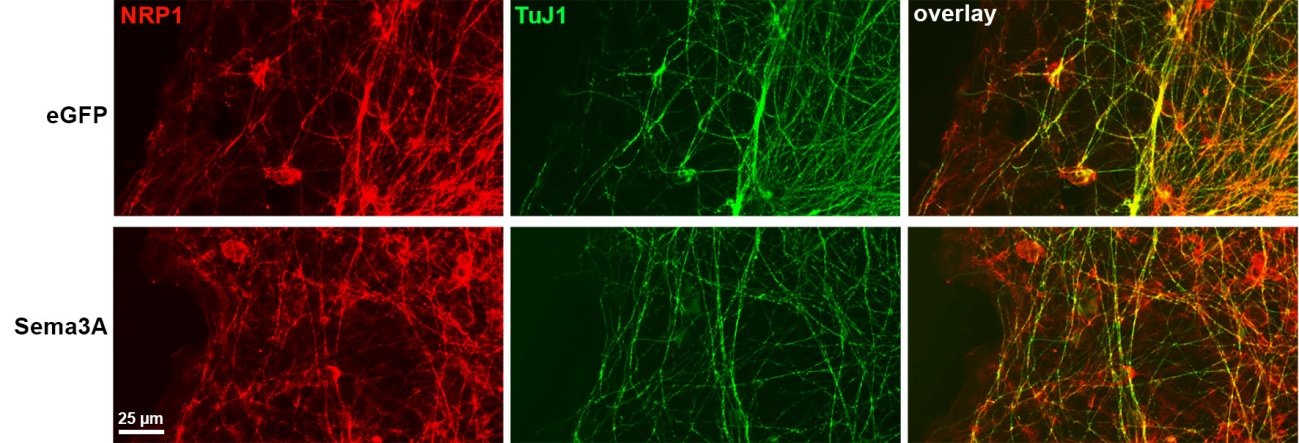

Supplement: Supplementary file 1 — Supplementary information [file 41598_2020_71865_MOESM1_ESM.docx]
